# Editorial: Immune infiltration and immunotherapy in cancer studies

**DOI:** 10.3389/fmolb.2024.1459242

**Published:** 2024-08-20

**Authors:** Udayan Bhattacharya, Pooja Chauhan, Manish Goyal

**Affiliations:** ^1^ Weill Cornell Medicine, Department of Pathology and Laboratory Medicine, New York, NY, United States; ^2^ Department of Radiation Oncology, Massachusetts General Hospital and Harvard Medical School, Boston, MA, United States; ^3^ Department of Molecular and Cell Biology, Boston University Goldman School of Dental Medicine, Boston, MA, United States

**Keywords:** immunotherapy, immune infiltration, tumor microenvironment (TME), pan-cancer analysis, cancer prognosis, biomarker analysis etc

**FIGURE 1:**
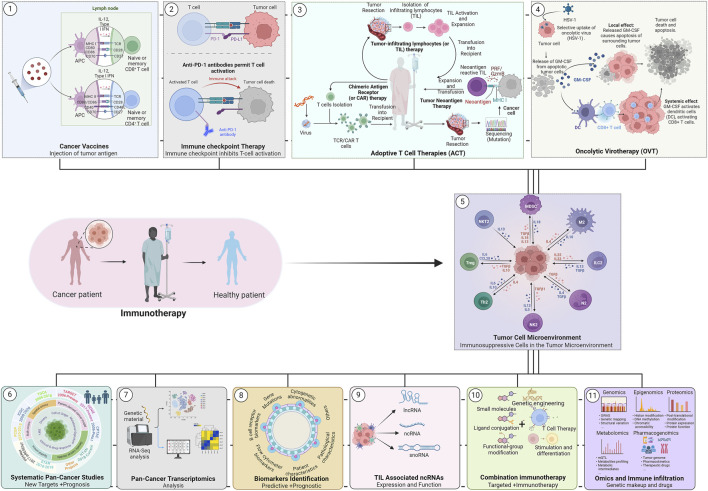
Schematic overview of the cancer immunotherapies. A wide array of cancer immunotherapy approaches has proven effective in human cancer patients, including the use of (1) Therapeutic/Preventive, cancer vaccines, (2) Immune checkpoint inhibitors therapy, and (3) Adoptive T cell therapies (ACT) and (4) Oncolytic virotherapy. Nonetheless, a deep understanding of the (5) Immune Microenvironment (TIME) is vital for designing an effective immunotherapy. In recent years, significant advancement was made in the field with the help of modern molecular genetic technologies that explored linked pathways and molecular mechanisms in human cancers including, (6) systematic pan-cancer analysis for prognosis and target identification, (7) Pan-cancer transcriptomics and immune infiltration analyses, (8) predictive and prognostic biomarkers analysis to assess the response to therapies, (9) tumor- /immune infiltration associated ncRNA (LncRNA, snoRNA, etc.), (10) combining immunotherapy and targeted therapies in various cancer treatments, and (11) multi-omics analysis of immune infiltration and immune microenvironments.

Cancer is a heterogeneous and multifactorial disease that accounts for millions of deaths each year (Bray et al., 2024; Swanton et al., 2024). Over the decades, a significant advancement was made in understanding the molecular mechanism(s) that cause the disease progression, leading to the development of several advanced therapeutic regimes (Pucci et al., 2019). Among them, immunotherapy has evolved as one of the revolutionized treatments against malignant tumors in extending patients’ disease-free survival thereafter (Waldman et al., 2020). At present, several different cancer immunotherapies are routinely used in clinical practice, including, 1) cancer vaccines, 2) immune checkpoint inhibitors (ICIs), 3) adoptive cell transfer (ACT), and 4) oncolytic virotherapy with great efficacy (Figure 1) (Yang et al., 2022; Rui et al., 2023). All these forms of immunotherapy have shown robust success in clinical settings; however, their efficacy varies from patient to patient. It has been observed that only a subset of cancer patients demonstrate durable responses to immunotherapy while the majority fail. The key reason behind the limited efficacy of immunotherapy is the heterogeneity of the tumor microenvironment (TME) and its elements among different patients and tumors. TME components interact with each other and shape an immunosuppressive microenvironment essential for tumor development (Figure 1) (de Visser and Joyce, 2023). Infiltrating immune cells are a crucial component of the TME and the correlation between them and tumor development can yield important biomarkers to support patient prognosis, and their response to immunotherapy (Fridman et al., 2011; de Visser and Joyce, 2023). Thus, tumor immunotherapy success largely depends on deep knowledge of the tumor immune microenvironment and its impact on response to immunotherapy. The current Research Topic focuses on recent advancements in immune infiltration and immunotherapy in various cancers, particularly addressing systematic pan-cancer studies, biomarker analysis, and combination therapy. Overall, the Research Topic includes five original research articles, 2 case reports, and one review article.

The expansion of whole genome sequencing and genome-wide expression studies across large datasets of human cancers presented the opportunity to undertake systematic approaches to dissect the shared genetic and transcriptional basis in different humancancers. In this regard, systematic pan-cancer studies using large datasets may play a pivotal role in identifying predictive biomarkers and multifaceted characteristics of oncogenes (Figure 1) (Consortium, 2020; Ganini et al., 2021). In this Research Topic, several leading groups contributed original research articles that emphasize the role of genome-wide systematic pan-cancer studies in tumorigenesis and identifying novel, unexplored biomarkers in various human cancers. The authors used multidimensional databases (TCGA, GTEx, and GEO, etc.) to uncover the association between oncogenes expression and cancer driver events, such as genetic alteration, protein phosphorylation, tumor mutative burden, microsatellite instability, DNA methylation, immune infiltration, and tumor immune microenvironment. The key oncogenes identified in different studies include *FUBP1*(far upstream element-binding protein 1) (Wang et al.), *CASP3* (gene encoding specific protease caspase 3) (Zhou et al.), *Lymphatic antigen 96* (*LY96,* involved in tumorigenesis by modulating host immunity) (Nie et al.), and *BATF* (transcription factor) (Jia et al.). The authors found a remarkable correlation of *FUBP1* and *CASP3* expression with cancer driver events, which suggested them as important biomarkers for cancer prognosis and immunotherapy in various human cancers (Wang et al.; Zhou et al.). Also, the author established an association between *LY96* expression and cancer-driven events, tumor microenvironment, and immune cell infiltration. The study supports the fact that *LY96* expression plays an important role in the prognosis of most cancers and is possibly involved in classic tumor-associated pathways and related to drug resistance (Nie et al.). Similarly, Jia et al. uncovered the role of *BATF* in immunotherapeutic and chemotherapy responses and reported its association with the survival of cancer patients (Jia et al.). Overall these studies envisaged the involvement of oncogenic proteins in immunotherapeutic responses in different cancers, thus, supporting a theoretical basis for targeting specific proteins in cancer immunotherapy.

Additionally, this Research Topic covers original research articles that highlight the importance of cancer biomarkers as molecular indicators for screening/early detection, diagnosis, prognosis, and response to immunotherapy. Mediastinal metastasis with unknown primary origin is a very rare condition characterized by poor prognosis with no known treatment. Biomarker analysis in a patient with squamous cell carcinoma with multiple lymph node metastases identified highly positive programmed cell death-ligand 1 (*PD-L1*) expression. Intriguingly, the patient responded well and has benefitted from 2-year survival by immunochemotherapy (anti-*PDL*-1 antibodies in combination with chemotherapy) (Zhao et al.). Likewise, Tang et al. identified Actin-related protein 2/3 complex subunit 1B (*ARPC1B*) as a prognostic biomarker for kidney renal clear cell carcinoma (KIRC) (Tang et al.). High *ARPC1B* expression is linked with poor overall survival (OS) and affects multiple immune-related functions. Altogether, these studies support the crucial role of biomarker analysis in the selection of optimal treatment regimens and immunotherapy in later-line treatment. Combination therapy is nowadays considered a gold standard practice for cancer treatment. However, in the case of advanced chromophobe renal cell carcinoma (ChRCC), the use of combination therapy is rare. In a recent case report, Zhang et al. showed that a combination of sintilimab (immunotherapy) with axitinib (targeted therapy) as a second-line treatment, greatly improved the metastatic lesions in the lungs. Overall, this study opened new avenues for a potential therapeutic option for patients with metastatic ChRCC. Finally, the only review article on this Research Topic contributed by Knight et al., (2023) focused on immunotherapy in melanoma treatment (Knight et al., 2023). Briefly, the article discussed the current treatments, ongoing immunotherapeutic and clinical strategies, pitfalls, and future perspectives for the advanced treatment of metastatic melanoma.

The hallmark of cancer progression and response to immunotherapy largely depends on a comprehensive, in-depth, and interconnected understanding of the heterogeneous tumor immune microenvironment landscape. The current Research Topic aims to broaden our understanding of different aspects of tumor immune microenvironment, immune cell infiltration pattern, and their association with tumor immunotherapy to uncover new therapeutic targets. We believe that the readers will find the articles in this Research Topic as useful references that will strengthen readers’ knowledge of the emerging field of cancer immunotherapy.
